# Fasting hyperglycaemia and fatty liver drive colorectal cancer: a retrospective analysis in 1145 patients

**DOI:** 10.1007/s11739-024-03596-6

**Published:** 2024-04-26

**Authors:** Lucilla Crudele, Carlo De Matteis, Fabio Novielli, Stefano Petruzzelli, Ersilia Di Buduo, Giusi Graziano, Marica Cariello, Elena Piccinin, Raffaella Maria Gadaleta, Antonio Moschetta

**Affiliations:** 1 Department of Interdisciplinary Medicine, University of Bari “Aldo Moro”, Piazza Giulio Cesare n. 11, 70124 Bari, Italy; 2 Center for Outcomes Research and Clinical Epidemiology (CORESEARCH), 65124 Pescara, Italy; 3 Department of Translational Biomedicine and Neuroscience (DiBraiN), University of Bari “Aldo Moro”, Bari, Italy; 4 INBB National Institute for Biostructure and Biosystems, Viale delle Medaglie d’Oro 305, 00136 Rome, Italia

**Keywords:** MASLD, Metabolic syndrome, Liver steatosis, Liver fibrosis, Colorectal cancer

## Abstract

**Background:**

Metabolic dysfunction-associated steatotic liver disease (MASLD) represents the hepatic manifestation of increased adiposopathy, whose pathogenetic features have been proposed as tumourigenic triggers for colorectal cancer (CRC). We aim to identify specific metabolic signatures involved in CRC development that may be used as non-invasive biomarkers, paving the way for specific and personalized strategies of CRC prevention and early detection.

**Methods:**

We retrospectively assessed CRC onset during a time frame of 8 years in a cohort of 1145 out-patients individuals who had previously been evaluated for Metabolic Syndrome.

**Results:**

28 patients developed CRC. No association between CRC development and visceral and general obesity was detected, while baseline fasting plasma glucose (FPG) and non-invasive liver fibrosis scores were significantly higher in patients with CRC, compared to those who did not develop cancer. Liver steatosis and MASLD were more frequently diagnosed in patients who developed CRC compared to no cancer developers. Canonical correlations among metabolic biomarkers were not present in CRC developers, differently from no cancer group. In ROC analysis, FPG and non-invasive scores also showed good sensitivity and specificity in predicting colon cancer. We then calculated ORs for metabolic biomarkers, finding that higher FPG and non-invasive scores were associated with an increased risk of developing CRC.

**Conclusion:**

MASLD and increased FPG may play a role in the clinical background of CRC, bringing to light the fascinating possibility of a reversed gut–liver axis communication in the pathogenesis of CRC. Thus, the use of non-invasive scores of fatty liver may be helpful to predict the risk of CRC and serve as novel prognostic factors for prevention and therapeutic strategies.

**Supplementary Information:**

The online version contains supplementary material available at 10.1007/s11739-024-03596-6.

## Introduction

Colorectal cancer (CRC) is one of the most common malignancies in the Western world [1], ranking as the second leading cause of cancer-related death [2]. Although colonoscopy represents the gold standard for CRC screening and diagnosis, the identification of novel blood-based biomarkers for CRC prediction are welcome since they would be minimally invasive and easily accepted by patients. Indeed, one challenge clinicians face is to identify patients at high risk, as they would benefit from peculiar screening and follow-up [3]. Recently, a growing body of evidence highlighted how tumour onset and progression is a synergistic process in which different players give a substantial contribution, pointing at obesity as the strongest risk factor contributing to global cancer burden [4].

Also CRC may be considered an obesity-related cancer [5] and multiple mechanisms are believed to drive this association, including hyperinsulinemia, inflammation, and dyslipidemia [6] with a direct role of hyperinsulinemia, more strongly than other aspects of Metabolic Syndrome (MetS) [7]. Patients with hyperinsulinemia are susceptible to pro-inflammatory state, that has been shown to promote mutagenesis, tissue damage, and ultimately carcinogenesis [8], and pro-thrombotic state that promotes colon cancer growth through platelet hyperactivity [9]. Also the hepatic manifestation of the multisystem disorder linked to increased visceral adipose tissue, defined as Metabolic dysfunction-associated steatotic liver disease (MASLD), has been proposed as risk factor for CRC [10–12]. An umbrella review of meta-analysis and observational studies have shown a 50% increased incidence of CRC in patients with diabetes [13], and according to a recent systematic review evaluating 14 studies, the incidence of CRC was 1.43 per 1000 person-years in patients with MASLD [14] that may drive CRC at younger age, compared to subjects without fatty liver [14]. When investigating cancer prevalence in MASLD patients, CRC was among the most frequently reported, and MASLD was associated with an increase in cancer-related mortality [15]. The disease process in MASLD is best described by the grade of activity and the stage of liver fibrosis [16], rather than a dichotomous classification (steatosis vs. steatohepatitis), and patients with advanced fibrosis seems to have a more significant association to increased CRC risk [17]. Therefore, the assessment and staging of liver fibrosis may help identifying high-risk subgroup for CRC among people with hepatic steatosis. Yet, fibrosis can be assessed only through liver biopsy and elastography that, similarly to colonoscopy, are not feasible as wide-spread examinations, considering biopsy invasiveness, the high prevalence of MASLD, and the need for monitoring its progression. In this context, several easy-to-use non-invasive tests (NITs) have been proposed as bio-humoral and clinical surrogates and have been recommended by clinical guidelines in individuals with fatty liver and suspected advanced fibrosis to perform appropriate risk stratification and predict morbidity and mortality [18]. Furthermore, their values also showed a stronger association with specific types of cancer [19].

For these reasons, the goal of this study was to identify specific metabolic signatures involved in CRC development that could be used as non-invasive biomarkers, paving the way for specific and personalized strategies of CRC prevention and early detection in patients with fatty liver.

## Materials and methods

### Study design and patient involvement

Between January and March 2023, we conducted telephonic interviews to retrospectively record the onset of CRC in 1266 individuals who had been previously evaluated for MetS at Internal Medicine Division “C. Frugoni” of University Hospital of Bari, Italy in the period between January 2015 and December 2020, excluding those with viral hepatitis and alcohol-related liver disease. When patients reported cancer diagnosis, we confirmed CRC by retrieving biopsy results directly from the hospital archival files, and when we could not find such data, we invited study participants to hand in their medical documentation.

They had been assigned a unique ID and registered in the electronic health register of Metabolic Diseases of the Department of Interdisciplinary Medicine at “Aldo Moro” University of Bari. After excluding those who had been diagnosed with other cancer types (*n* = 121), the final analysis was performed on a population of 1145 subjects. At the time of enrolment, this study (n.311, MSC/PBMC/2015) was approved by the Ethics Committee of the Azienda Ospedaliero-Universitaria Policlinico di Bari (Bari, Italy), in accordance with the requirements of the Declaration of Helsinki. Written informed consent for the use of clinical data was obtained from all participants in this study. In accordance with the approved Ethics Committee, only patients who were already 18 years old or more were included.

### Data collection

At the time of enrolment, a standardized questionnaire collecting detailed information on reproductive history, smoking and alcohol drinking history, exposure to environmental toxics, medical history, educational level, and other socioeconomic and lifestyle variables was administered to patients (Supplementary Table 1). Information on drug use, occupation and family history of cancer had also been collected. Moreover, physical examination, anthropometric measures, biochemical assessment, and abdomen ultrasound to detect liver steatosis were performed. After an overnight fasting, patients underwent an abdominal ultrasound scanning performed by two expert physicians with more than 10 years of experience in ultrasonography with a 3.5–5 MHz convex probe (Esaote MyLab 70 Gold ultrasound system) [20].

Anthropometric assessment was performed using standardized procedures. Briefly, waist circumference (WC) was measured at the midpoint between the inferior part of the 12th costa and the anterior–superior iliac crest. Body Mass Index (BMI) was computed as weight (Kg) divided by the height squared (sqm) and BMI values (Kg/sqm) between 25 and 29.9 and over 30.0 were considered as overweight and obesity conditions, respectively. MetS was diagnosed according to International Diabetes Federation (IDF) definition [21] and visceral obesity was defined for WC values above 80 cm in women and 94 cm in men. Type 2 Diabetes (T2D) was diagnosed when glycosylated haemoglobin (HbA1c) ≥ 48 mmol/mol and/or fasting plasma glucose (FPG) ≥ 126 mg/dl and/or ongoing treatment for diabetes, while prediabetes was defined as impaired fasting glycaemia (100 < FPG < 126) and/or 42 < HbA1c < 48. MASLD diagnosis was based on the presence of liver steatosis identified by ultrasound and at least one of the five criteria for MetS, also considering BMI ≥ 25 kg/sqm to assess overweight or obesity alternatively to increased WC [22].

Non-invasive scores were determined according to published formulas (see Supplementary Table 2). Specifically, we calculated AST to ALT ratio (AAR), AST to Platelet Ratio Index (APRI); (AST to ALT ratio) to platelet ratio index (AARPRI), BMI, ALT, Age, and Triglycerides (BAAT); Fibrosis-4 Index (FIB-4); modified FIB-4 (mFIB-4), Fatty Liver Index (FLI), Hepatic Steatosis Index (HIS), NAFLD-Liver Fat Score (NAFLD-LFS), NAFLD Fibrosis Score (NFS), BARD score, Forns Score, and NFS-Ridge score.

Morning blood samples were obtained after 12 h of fasting from the antecubital veins, and then biochemical markers of glucose and lipid metabolism were measured in patients’ serum. After blood clotting and centrifugation, serum was processed, and liver and thyroid markers were measured following standardized biochemical procedures. All biochemical measurements were centralized and performed in the ISO 9001 certified laboratories of the University Hospital of Bari.

### Data analysis

Descriptive statistical analyses of the study sample were performed, and results are expressed as mean ± standard deviation (SD) for numerical data, in counts and percentages for categorical data. Comparisons of clinical variables between two groups were conducted with Mann–Whitney *U* Test, while comparisons between categorical variables were performed with chi-squared test. To investigate the role of possible confounders, we performed analysis of covariance (ANCOVA) and computed the *T*-test for the difference between group means adjusted for the covariate. All reported *p*-values (*p*) were based on two-sided tests and compared to a significance level of 5%.

Empirical ROC curves were plotted along with a calculation of the area under the curve (AUC) to give us a measure of the capability to distinguish between CRC and no cancer groups and Youden’s Index (YI), or equivalently, the highest sensitivity + specificity, was used to determine the optimal cut-off.

Chi-square test, along with Fisher’s exact test if indicated, was used to study the association between categorical variables and Odds Ratio (OR) with their relative 95% confidence interval (95% CI) was calculated. Correlations among variables were also analysed and estimated using Spearman correlation coefficient (r). All analyses were performed using the NCSS 2023 Statistical Software (2023, NCSS, LLC. Kaysville, Utah, USA) and GraphPad Prism, version 10 (GraphPad Software; San Diego, CA, USA).

## Results

### Baseline characteristics of study population

Out of 1145 subjects (538 males, 607 females), 28 (2.45%; 22 males, 6 females) were diagnosed with CRC during the last eight years.

Mean age of population sample was 56.1 ± 15 years. WC (97.8 ± 14.7) was above the established cut-off for MetS diagnosis, as along with a mean BMI (27.3 ± 5.8) depicting a condition of overweight. When considering bio-humoral values, we found that mean FPG was 101.3 ± 29.9 and HbA1c was 41.6 ± 12.5 depicting an overall condition of prediabetes. Liver steatosis was US detected in 525 individuals and MASLD was diagnosed in 495 of them.

Table 1 summarizes all baseline characteristics of the population.

**Table 1:** Characterization of the study population (*n* = 1145)

Sex (M:F)	538:607
Age (years)	56.1 ± 15
Waist Circumference (cm)	97.8 ± 14.7
BMI (Kg/sqm)	27.3 ± 5.8
FPG (mg/dL)	101.3 ± 29.9
HbA1c (mmol/mol) (*n* = 798)	41.6 ± 12.5
AST (U/L)	23.5 ± 13.2
ALT (U/L)	30.9 ± 17.5
GGT (U/L)	34.5 ± 35.6
ALP (U/L)	69.9 ± 22.2
Total cholesterol (mg/dL)	183.6 ± 42.5
HDL cholesterol (mg/dL)	54.1 ± 15.5
LDL cholesterol (mg/dL)	105.5 ± 34.6
Triglycerides (mg/dL)	120.2 ± 74.5

Data are reported as mean ± SD (Standard Deviation)

*BMI* Body Mass Index, *FPG* Fasting Plasma Glucose, *HbA1c* glycosylated haemoglobin, *ALP* Alkaline Phosphatase

### Biomarkers comparisons between CRC and control groups

We then performed a comparison between clinical and biochemical features of patients who developed CRC and did not (Table 2). Patients who developed CRC were older (*p* < 0.05) and presented baseline increased FPG (*p* < 0.005) and glycosylated haemoglobin (HbA1c) (*p* < 0.05) compared to patients who did not develop CRC. Liver transaminases AST (*p* < 0.01) and GGT (*p* < 0.05) were significantly increased in patients who developed CRC, while no significant differences were detected in ALT and Alkaline Phosphatase (ALP) levels as well as in lipid profile.

**Table 2:** Comparison of clinical and bio humoral variables between patients who developed colorectal cancer (CRC) and did not

	No cancer (*n* = 1117, 516 M:601F)	CRC (*n* = 28, 22 M:6F)	*p*-value
Age (years)	55.9 ± 15.1	62.9 ± 9.5	< 0.05
Waist circumference (cm)	97.7 ± 14.7	100.2 ± 12.3	ns
BMI (Kg/sqm)	27.3 ± 5.9	27.8 ± 4.3	ns
FPG (mg/dL)	100.8 ± 29.1	119.5 ± 49.8	< 0.005
HbA1c (mmol/mol)	41.4 ± 12.2	47.8 ± 20.5	< 0.05
AST (U/L)	23.3 ± 12.7	33.8 ± 24.4	< 0.01
ALT (U/L)	30.8 ± 17.2	37.8 ± 25.8	ns
GGT (U/L)	34.3 ± 35.6	43.2 ± 35.7	< 0.05
ALP (U/L)	69.9 ± 22.2	71.6 ± 25	ns
Total cholesterol (mg/dL)	183.6 ± 42.4	184.2 ± 44.3	ns
HDL cholesterol (mg/dL)	54.1 ± 15.3	53.4 ± 22.1	ns
LDL cholesterol (mg/dL)	105.5 ± 34.6	104.7 ± 35.8	ns
Triglycerides (mg/dL)	119.6 ± 73.7	141.9 ± 98.6	ns
US Liver Steatosis (*n*; %)	506 (45)	19 (68)	< 0.05
MASLD (*n*; %)	476 (43)	19 (68)	< 0.01
Type 2 Diabetes (*n*; %)	403 (36)	14 (50)	ns
Impaired Fasting Glycaemia (*n*; %)	100 (9)	3 (11)	ns
Smoking (*n*; %)	369 (33)	12 (43)	ns

Data are reported as mean ± SD (Standard Deviation). Comparisons between patients with and without colorectal cancer were performed by Mann–Whitney test for continuous variables and with Chi-squared test for categorical variables; *p*-value < 0.05 was considered significant

*M* males, *F* females, *BMI* Body Mass Index, *FPG* Fasting Plasma Glucose, *HbA1c* glycosylated haemoglobin, *ALP* Alkaline Phosphatase, *US* ultrasound, *MASLD* Metabolic dysfunction-associated steatotic liver disease, *ns* not significant

We compared the presence of US liver steatosis in the two groups, finding that its proportion was significantly greater (*p* < 0.05) in CRC developers (68%) compared to no cancer group (45%). When considering MASLD, this difference was even more pronounced (*p* < 0.01) since all CRC developers with liver steatosis had also MASLD, while this was not true in subjects who did not develop CRC (43%), thus suggesting that an association with CRC development could be found for fatty liver and MASLD.

In respect of the significant differences regarding glycaemic profile, we then considered T2D and impaired fasting glycaemia conditions, finding that nor the first neither the second showed a significant association with CRC development, although a trend could be identified. We finally investigated if another risk factor as smoking habits could interfere or having an adding weight in colorectal carcinogenesis. Also in this case, we could not identify a significant difference between the two groups.

**Fig. 1:**
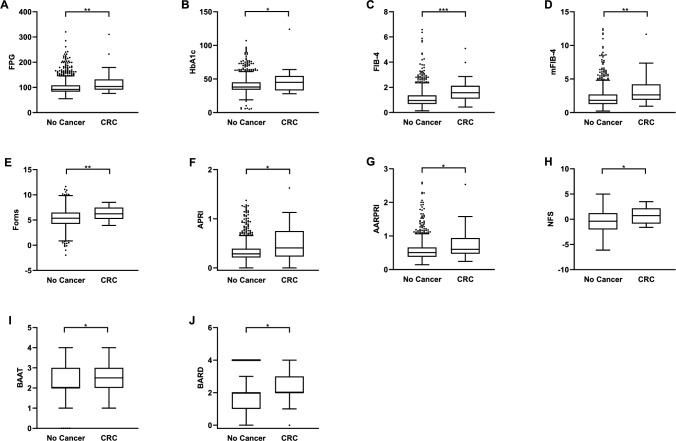
Variables which were significantly increased in patients who later developed colorectal cancer (CRC). Comparisons were performed by Mann–Whitney test and *p*-value < 0.05 was considered significant. The box plots show the median (second quartile), first and third quartile, and whiskers represent minimum and maximum values. *p < 0.05; **p < 0.005; ***p < 0.0001. *FPG* Fasting plasma glucose, *HbA1c* glycosylated haemoglobin, *FIB-4* Fibrosis-4 index, *mFIB-4* modified FIB-4, *APRI* AST to Platelet Ratio Index, *AARPRI* (AST to ALT ratio) to Platelet Ratio Index, *NFS* NAFLD Fibrosis Score; *BAAT* BMI, ALT, Age, and Triglycerides

Considering NITs, FIB-4 (1.1 ± 0.7 vs 1.8 ± 1, *p* < 0.0001), mFIB-4 (2.2 ± 1.5 vs 3.3 ± 2.3, *p* < 0.001), FORNS (5.3 ± 1.8 vs 6.3 ± 1.3, *p* < 0.005), APRI (0.3 ± 0.2 vs 0.5 ± 0.4, *p* < 0.05), AARPRI (0.6 ± 0.3 vs 0.8 ± 0.5, *p* < 0.05), NFS (− 0.4 ± 2.1 vs 0.8 ± 1.6, *p* < 0.01), BAAT (2.1 ± 0.7 vs 2.5 ± 0.6, *p* < 0.01), and BARD (1.7 ± 1.2 vs 2.3 ± 1, *p* < 0.01) were found significantly increased in CRC group (Fig. 1). Conversely, there were no significant differences between the two groups regarding AAR (0.8 ± 0.3 vs 1 ± 0.5, p = ns), FLI (48.1 ± 31.5 vs 58.9 ± 30.2, *p* = ns), NFS-Ridge (− 5.9 ± 5.3 vs − 4.5 ± 7.9, *p* = ns), and HSI (35.7 ± 6.5 vs 37 ± 4.3, *p* = ns).

Considering the significant difference in age between the two groups, we then performed an age-adjusted comparison for NITs, finding that FIB-4, APRI, ARRPRI, and BAAT still significantly distinguished between CRC developers and not-developers (Table 3).

**Table 3:** Age-adjusted comparison of NITs between patients who developed colorectal cancer (CRC) and did not

	No cancer (*n* = 1117, 516 M:601F)	CRC (*n* = 28, 22 M:6F)	*p*-value
FIB-4	1.1 ± 0.1	1.6 ± 0.1	0.001**
mFIB-4	2.2 ± 0.1	2.7 ± 0.3	ns (0.108)
FORNS	5.3 ± 0.1	5.8 ± 0.3	ns (0.082)
APRI score	0.3 ± 0.1	0.5 ± 0.1	0.0001***
AARPRI score	0.6 ± 0.1	0.7 ± 0.1	0.036*
NFS	− 0.4 ± 0.1	0.3 ± 0.5	ns (0.132)
BAAT score	2.1 ± 0.1	2.5 ± 0.1	0.008*
BARD score	1.7 ± 0.1	2.0 ± 0.3	ns (0.377)

Analysis of covariance (ANCOVA) and the *T*-test for the difference between group means adjusted for age as covariate was performed. *p*-value < 0.05 was considered significant. *p < 0.05; **p < 0.001; ***p < 0.0001

*BMI* Body Mass Index, *FPG* Fasting Plasma Glucose, *HbA1c* glycosylated haemoglobin, *ALP* Alkaline Phosphatase, *FIB-4* Fibrosis-4 Index, *NFS* NAFLD Fibrosis Score

#### Different correlations among metabolic biomarkers in CRC and control groups

**Fig. 2:**
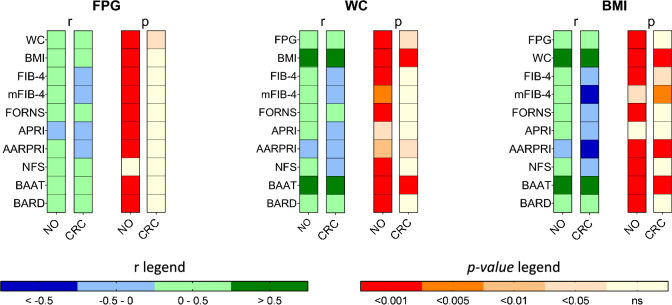
Correlations of fasting plasma glucose, waist circumference and BMI with non-invasive scores of fatty liver. Heatmaps show Spearman correlations (*r*) among FPG, WC, BMI, and non-invasive scores of fatty liver in patients with (CRC) and without (NO) colorectal cancer and corresponding *p*-values (*p*). *FPG* Fasting Plasma Glucose, *BMI* Body Mass Index, *WC* Waist Circumference, *FIB-4* Fibrosis-4 index, *mFIB-4* modified FIB-4, *APRI* AST to Platelet Ratio Index, *AARPRI* (AST to ALT ratio) to Platelet Ratio Index, *NFS* NAFLD Fibrosis Score, *BAAT* BMI, ALT, Age, and Triglycerides, *ns* not significant. *p* < 0.05 were considered significant

Considering the significant increase in their values, we then tried to deepen correlations among FPG and NITs in cancer and control groups. While in no cancer population, canonical correlations of FPG with non-invasive scores were detected, these correlations were not shown in CRC population. Also, when considering anthropometric parameters such as WC and BMI, we found that their well-known relationships with glycaemia and NITs were already lost at baseline in patients who were later diagnosed with CRC (Fig. 2).

### Risk of developing CRC

**Fig. 3:**
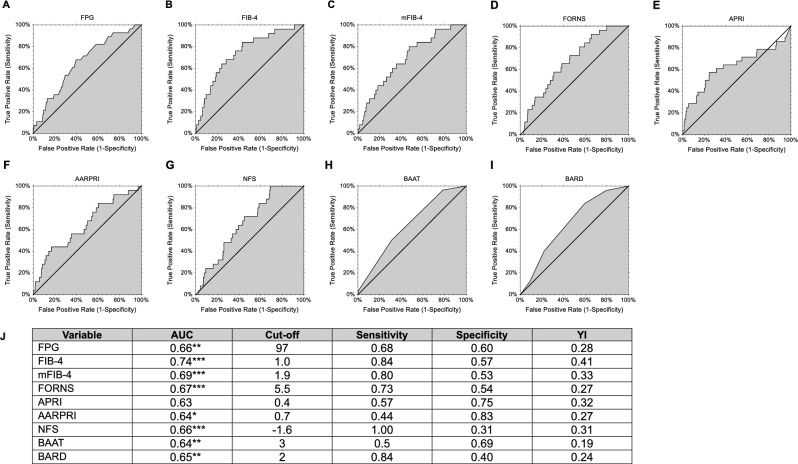
ROC curves for colorectal cancer developers detection. ROC curves for detection of patients who later developed colorectal cancer are shown for FPG (**A**), FIB-4 (**B**), mFIB-4 (**C**), FORNS (**D**), APRI (**E**), AARPRI (**F**), NFS (**G**), BAAT (**H**), BARD (**I**). In the table (**J**) empirical estimation of area under curve (AUC) with 95% confidence intervals and two-sided upper p-values for null hypothesis AUC = 0.5 (*p < 0.05; **p < 0.005; ***p < 0.001), cut-off values with related sensitivity and specificity levels, and Youden’s Index (YI) are reported. *FPG* Fasting Plasma Glucose, *FIB-4* Fibrosis-4 index, *mFIB-4* modified FIB-4, *APRI* AST to Platelet Ratio Index, *AARPRI* (AST to ALT ratio) to Platelet Ratio Index, *NFS* NAFLD Fibrosis Score, *BAAT* BMI, ALT, Age, and Triglycerides, *YI* Youden’s Index

With the aim of determining if the above-considered biomarkers could be associated ahead of time with CRC onset, we then performed ROC curves of those variables that had been found significantly different at baseline in the two groups (Fig. 3). FPG and all non-invasive scores, except APRI, showed a significant AUC. Specifically, FPG showed AUC = 0.66 (*p* < 0.005), with a cut-off value of 97 mg/dL, a sensitivity of 0.68 and a specificity of 0.60. When considering NITs, FIB-4 showed the highest AUC (0.74, *p* < 0.001) and YI (0.41, sensitivity 0.84, specificity 0.57) for a cut-off value of 1.

**Fig. 4:**
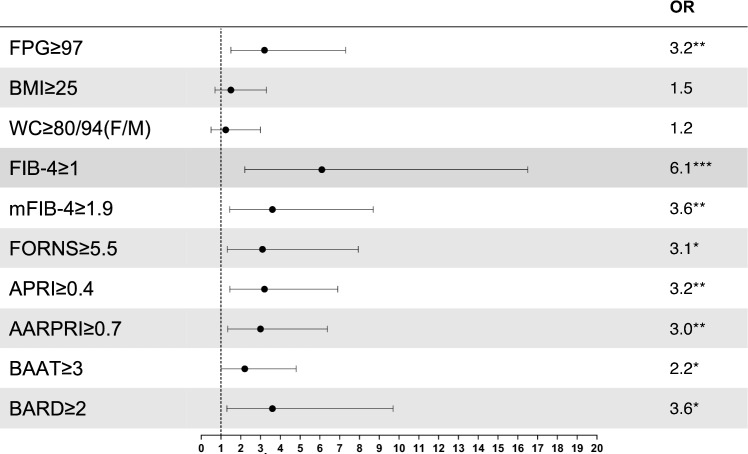
Risk of developing colorectal cancer in subjects with increased metabolic and anthropometric variables. Forest plot represents Odds Ratios (OR) with their relative 95% confidence interval (CI). Chi-squared test, along with Fisher’s exact test if indicated, was used to study the association between CRC and increased FPG, non-invasive liver fibrosis scores and anthropometric measures. *p < 0.05; **p < 0.005; ***p < 0.001. *FPG* Fasting Plasma Glucose, *WC* Waist Circumference, *BMI* Body Mass Index, *M* males, *F* females, *FIB-4* Fibrosis-4 index, *mFIB-4* modified FIB-4, *APRI* AST to Platelet Ratio Index, *AARPRI* (AST to ALT ratio) to Platelet Ratio Index, *NFS* NAFLD Fibrosis Score, *BAAT* BMI, ALT, Age, and Triglycerides

To assess the risk of developing CRC when baseline FPG or NITs are increased, we then calculated OR (Fig. 4), finding that all non-invasive scores showed a significant association with CRC (*p* < 0.05). FIB-4, that had already shown the best YI in ROC analysis, showed the highest OR (6.1, 95% CI 2.2–16.5, *p* < 0.001). Regarding FPG, the risk of getting CRC for a subject with increased FPG at baseline was more than three times higher than a subject with glycaemia levels under 97 mg/dL (OR = 3.2, 95% CI 1.5–7.3; *p* < 0.01). Finally, we also calculated OR for BMI and WC to determine whether the international cut-offs for overweight and visceral obesity are consistent with an increased CRC risk, but no association was found.

## Discussion

In this study, we investigated whether some non-invasive metabolic biomarkers are associated with the risk for CRC, as a consequence of the pathogenetic involvement of fatty liver infarction in its pathogenesis.

In individuals who later developed CRC, we found increased baseline fasting glycaemia and HbA1c, showing that subjects with higher FPG had more than three times the risk of developing CRC. Since mean values of such parameters depicted a condition of diabetes, also the hypothesis that chronic inflammation and hyperinsulinemia may act as direct carcinogenetic factors should be considered. However, the evidence that canonical relationship between FPG and fatty liver scores are lost in patients who later develop CRC suggests that hyperglycaemia and insulin resistance should not be considered as stand-alone triggers for CRC but in the context of the broad metabolic derangements associated to unhealthy lifestyle [23]. Since exacerbated fatty acid metabolism is an additional lipid signature of tumour cells [24], the derangement of canonical correlations among biochemical markers in our CRC group may mirror the uncoupling of cholesterol and lipid metabolism that are important drivers of tumour progression [25, 26]. Different microenvironmental factors, such as those inducing endoplasmic reticulum stress, promote fatty acid synthesis and dampen immune responses [27]. Similarly to hepatocytes, cancer cells increase the uptake of dietary free fatty acids and lipoproteins or upregulate the endogenous lipogenesis and cholesterol synthesis to answer the high lipid and cholesterol demands, necessary to sustain endlessly proliferating cells. These pathways contribute to invasion and metastasis through transcriptional and epigenetic changes, also promoting inflammation in the tumour microenvironment, and avoiding immune destruction [28]. Consequently, in patients with subsequent CRC development, the baseline levels of cholesterol and triglycerides could result normal because they are already fuelling cancer cells growth, as also supported by a previous study in which no associations between CRC risk and HDL-c and LDL-c levels were detected [29].

The not-significant association we found between obesity (assessed through both BMI and WC) and CRC development may apparently contrast with the evidence about adiposopathy role in cancer development and mortality [30, 31]. It is important to point out that values of WC registered in our cohort were barely above the IDF cut-offs for MetS and that mean BMI values depict a condition of overweight but not obesity in both no cancer and CRC group. Moreover, also the possible weight loss due to underdiagnosed CRC cases at baseline should be considered among reasons of such inconsistency.

On the other hand, we also propose another fascinating scenario to explain these apparently paradoxical findings. Since alterations of cholesterol metabolism influence tumour cells and increased cholesterol biosynthesis also promotes inflammation [32], this reprogramming of metabolic pathways could be not a consequence of cancer, but the reason for CRC development, eventually anticipating carcinogenesis. Indeed, NITs are markers of hepatocytes fat infarction that, acting as triglycerides depot, may explain why no differences were found between our two groups in circulating triglycerides levels, despite the increase of NITs in CRC developers.

In this context, higher incidence in economically developed countries compared to developing countries highlights the crucial role of diet in determining CRC onset [33] as the major modifiable risk factor [34, 35]. Among the pro- and antitumoural dietary factors, lipids appear to be of crucial importance since high-fat diet (HFD) consumption, especially with high saturated fatty acids (SFA) content, favours hyperproliferation of intestinal stem cells [36] while the supplementation with oleic acid, the predominant fatty acid component of olive oil, reduces intestinal inflammation and tumour development [37]. In the liver, HFD results in an altered saturated to monounsaturated fatty acid (MUFA) ratio, with the concomitant decreased de novo lipogenesis programmes and increased fatty acids β-oxidation pathways, through the suppression of stearoyl CoA desaturase 1 (SCD1) [38].

Furthermore, HFD also induces alterations in the microbial profile and bile acid metabolism [39] that encourage tumourigenesis. While diet shapes the gut microbiota, probably on the basis of selection of bacteria species as dictated by the quantity and quality of ingested nutrients [40], a vicious circle is established by which specific gut bacteria affect carbohydrate metabolism and are associated with insulin resistance [41] and metabolic changes that alter the cancer metabolome to create conditions conductive for normal to cancer cell transformation [42]. That is why we also considered MASLD, the liver manifestation of metabolic diseases and its non-invasive scores for the assessment of CRC risk, thus paving the way to propose fatty liver as the dominant common link driving CRC and obesity association.

This study presents some limitations that need to be discussed. First, since it is a retrospective study, it can only define an association but not a causal relationship between metabolic impairment and cancer development. Secondly, although NITs included in the study were created for different specific conditions (i.e. liver steatosis or fibrosis), we considered them as a whole mix to better encompass the wide clinical range of liver impairment that may occur in MASLD [43]. Finally, ROC analysis for the prediction of CRC showed AUC values which are not high enough to suggest a single test use in clinical practice. However, the aim of this study was to identify any condition that could highlight the need for further investigation and more frequent follow-up in at-risk patients and not to verify whether a single score could be useful as a standing-alone marker, since NITs should always be interpreted according to the clinical context and considering the results of other tests (biochemical, radiological, and endoscopic) [44]. Thus, we further underline that each NIT, along with fasting hyperglycaemia, should be considered in the context of the broad metabolic derangements associated to MASLD*.*

Here, we postulate a new paradigm pointing at the liver–gut axis in which hepatocytes inflammation due to fat infarction together with increased fasting glucose levels represent the *primum movens* of metabolic derangements leading to CRC development. Due to the intertwined relationship between the gut and liver, the gut may represent the first target of systemic metabolic impairment due to fatty liver disease through the action of several enterokines and hormones. Owing to our findings, it is crucial to properly diagnose MASLD to offer patients a more tailored follow-up and screening programmes to minimize the risk of CRC development and to strengthen the importance of healthy behaviours in cancer prevention.

### Supplementary Information

Below is the link to the electronic supplementary material.

Supplementary file1 (PDF 127 KB)

Supplementary file2 (PDF 170 KB)

## Declarations

### Data availability

Data presented in this study are available on request from the corresponding author.
