# Supplementary material for: Fasting hyperglycaemia and fatty liver drive colorectal cancer: a retrospective analysis in 1145 patients
Source: Intern Emerg Med. 2024 Apr 26;19(5):1267–77. doi: 10.1007/s11739-024-03596-6 (PMC11364717; doi:10.1007/s11739-024-03596-6)
Supplement: Supplementary file 1 — Supplementary file1 (PDF 127 KB) [file 11739_2024_3596_MOESM1_ESM.pdf]

SUPPLEMENTARY MATERIAL 1 – QUESTIONNAIRE ADMINISTERED TO ENROLLED INDIVIDUALS

PATIENT____

DATE OF EVALUATION____________________________OPERATOR _________________________________

WC_____ HC ____ NC ____ WEIGHT ____ HEIGHT_____

SMOKING	YES	NO	CIGARETTES PER DAY_____ HOW MANY YEARS______
CELIAC DISEASE	YES	NO	
BREAST FEEDING	YES	NO	
DYSLIPIDEMIA	YES	NO	
DIABETES	YES	NO	EV. YEAR OF DIAGNOSIS __________ FAMILIARITY__________
SAT OCCLUSIVE DIS.	YES	NO	GRADE ________________________
MACE	YES	NO	YEAR __________
LIVER DISEASE	YES	NO	○ ALCOHOL-RELATED ○ HBV ○ HCV
MICI	YES	NO	○ URC ○ CROHN DISEASE ○ EoC
THYROID DISEASE	YES	NO	SPECIFY__________________ FAMILIARITY____________
CANCER DIAGNOSIS<br>YEAR __________	YES	NO	SPECIFY DIAGNOSIS ______________________________<br>○ CHT ○ FAMILIARITY _______________<br>○ RT ○ FIRST DIAGNOSIS? ______________<br>○ SURGERY ○ FOLLOW-UP
BRISTOL SCALE:			

LACTOSE INTOLL.	YES	NO
NATURAL CHILDBIRTH	YES	NO
HYPERTENSION	YES	NO

PCOS	YES	NO
ATRIAL FIBRILLATION	YES	NO

SCREENING PROCEDURES	YEAR	RESULTS
LAST EGD		
LAST COLONOSCOPY		
LAST FIT		

**HOW FREQUENTLY DID YOU DRINK LAST YEAR?**	NONE	<1 per MONTH	2-4 per MONTH	2-3 per WEEK	>= 4 per WEEK
**HOW MANY ALCOHOL BEVERAGES DID YOU DRINK IN A DAY IN WHICH YOU TOOK ALCOHOL, IN THE LAST YEAR?**	1-2	3-4	5-6	7-9	>= 10
**HOW MANY TIMES, IN THE LAST YEAR, DID YOU DRINK 6 OR MORE ALCOHOLIC BEVERAGES IN THE SAME DAY?**	NONE	<1 per MONTH	MONTHLY	WEEKLY	DAILY
