# Supplementary material for: Fasting hyperglycaemia and fatty liver drive colorectal cancer: a retrospective analysis in 1145 patients
Source: Intern Emerg Med. 2024 Apr 26;19(5):1267–77. doi: 10.1007/s11739-024-03596-6 (PMC11364717; doi:10.1007/s11739-024-03596-6)
Supplement: Supplementary file 2 — Supplementary file2 (PDF 170 KB) [file 11739_2024_3596_MOESM2_ESM.pdf]

Supplementary table 2. Non-invasive scores for fatty liver with their associated formulas

Scores	Formulas
AAR (1)	AST/ALT
FIB-4 (2)	$\frac{\text{Age} \times \text{AST}}{\text{Platelet Count} \times \sqrt{\text{ALT}}}$
mFIB-4 (3)	$\frac{10 \times \text{Age} \times \text{AST}}{\text{Platelet Count} \times \text{ALT}}$
FORNS (4)	$7.811 - 3.131 \times ln(platelet\ count) + 0.781\ ln(GGT) + 3.467 \times ln(age) - 0.014\,(total\ cholesterol)$
APRI (5)	$\frac{\text{AST}/ULN\ of\ normal\ value \times 100}{\text{Platelet Count}}$
AARPRI (6)	$\frac{\text{AST/ALT} \times 150}{\text{Platelet Count}}$
FLI (7)	$e^y/(1+e^y) \times 100$<br>Where y = 0.953 × ln(triglycerides) + 0.139 × BMI, + 0.718 × ln (GGT) + 0.053 × waist circumference – 15.745
NFS (8)	-1.675 + (0.037×age) + (0.094×BMI) + (1.13×IFG or diabetes [yes = 1, no = 0]) + (0.99×AAR) – (0.013×platelet count) – (0.66 ×albumin)
NFS-RIDGE (9)	-0.614+0.007×ALT- 0.214×HDL-0.053×Triglycerides +0.144×HbA1+0.032×WBC+0.132×hypertension
BAAT (10)	BMI≥28, age≥50, ALT≥2xULN, triglycerides≥1.7 mmol
BARD (11)	BMI ≥ 28: No = 0, Yes = 1; AST/ALT ratio: ≥0.8 No = 0, Yes = 2; and Diabetes: No = 0, Yes = 1
HSI (12)	8 × ALT/AST + BMI (+2, if type 2 diabetes; +2, if female)

Abbreviations: AAR: AST to ALT Ratio; FIB-4: Fibrosis-4 index; mFIB-4: modified FIB-4; APRI: AST-Platelet Ratio Index; AARPRI: (AST to ALT ratio) to Platelet Ratio Index; FLI: Fatty Liver Index; NFS: NAFLD Fibrosis Score; BAAT: BMI, ALT, Age and Triglycerides; HSI: Hepatic Steatosis Index.

References

1. Botros M, Sikaris KA. The de ritis ratio: the test of time. Clin Biochem Rev. 2013 Nov;34(3):117–30.

2. Sterling RK, Lissen E, Clumeck N, Sola R, Correa MC, Montaner J, et al. Development of a simple noninvasive index to predict significant fibrosis in patients with HIV/HCV coinfection. Hepatol Baltim Md. 2006 Jun;43(6):1317–25.

3. Wang HW, Peng CY, Lai HC, Su WP, Lin CH, Chuang PH, et al. New noninvasive index for predicting liver fibrosis in Asian patients with chronic viral hepatitis. Sci Rep. 2017 Jun 12;7(1):3259.

4. Wai CT, Greenson JK, Fontana RJ, Kalbfleisch JD, Marrero JA, Conjeevaram HS, et al. A simple noninvasive index can predict both significant fibrosis and cirrhosis in patients with chronic hepatitis C. Hepatol Baltim Md. 2003 Aug;38(2):518–26.

5. Lin ZH, Xin YN, Dong QJ, Wang Q, Jiang XJ, Zhan SH, et al. Performance of the aspartate aminotransferase-to-platelet ratio index for the staging of hepatitis C-related fibrosis: an updated meta-analysis. Hepatol Baltim Md. 2011 Mar;53(3):726–36.

6. Tseng PL, Wang JH, Hung CH, Tung HD, Chen TM, Huang WS, et al. Comparisons of noninvasive indices based on daily practice parameters for predicting liver cirrhosis in chronic hepatitis B and hepatitis C patients in hospital and community populations. Kaohsiung J Med Sci. 2013 Jul;29(7):385–95.

7. Bedogni G, Bellentani S, Miglioli L, Masutti F, Passalacqua M, Castiglione A, et al. The Fatty Liver Index: a simple and accurate predictor of hepatic steatosis in the general population. BMC Gastroenterol. 2006 Nov 2;6:33.

8. Angulo P, Hui JM, Marchesini G, Bugianesi E, George J, Farrell GC, et al. The NAFLD fibrosis score: a noninvasive system that identifies liver fibrosis in patients with NAFLD. Hepatol Baltim Md. 2007 Apr;45(4):846–54.

9. Yip TCF, Ma AJ, Wong VWS, Tse YK, Chan HLY, Yuen PC, et al. Laboratory parameter-based machine learning model for excluding non-alcoholic fatty liver disease (NAFLD) in the general population. Aliment Pharmacol Ther. 2017 Aug;46(4):447–56.

10. Ratziu V, Giral P, Charlotte F, Bruckert E, Thibault V, Theodorou I, et al. Liver fibrosis in overweight patients. Gastroenterology. 2000 Jun;118(6):1117–23.

11. Harrison SA, Oliver D, Arnold HL, Gogia S, Neuschwander-Tetri BA. Development and validation of a simple NAFLD clinical scoring system for identifying patients without advanced disease. Gut. 2008 Apr 29;57(10):1441–7.

12. Lee JH, Kim D, Kim HJ, Lee CH, Yang JI, Kim W, et al. Hepatic steatosis index: a simple screening tool reflecting nonalcoholic fatty liver disease. Dig Liver Dis Off J Ital Soc Gastroenterol Ital Assoc Study Liver. 2010 Jul;42(7):503–8.
